# Functional characterization and analgesic effects of mixed cannabinoid receptor/T-type channel ligands

**DOI:** 10.1186/1744-8069-7-89

**Published:** 2011-11-17

**Authors:** Haitao You, Vinicius M Gadotti, Ravil R Petrov, Gerald W Zamponi, Philippe Diaz

**Affiliations:** 1 Department of Physiology and Pharmacology, Hotchkiss Brain Institute, University of Calgary, Calgary, Canada; 2 Core Laboratory for Neuromolecular Production, The University of Montana, Missoula, MT, USA

## Abstract

**Background:**

Both T-type calcium channels and cannabinoid receptors modulate signalling in the primary afferent pain pathway. Here, we investigate the analgesics activities of a series of novel cannabinoid receptor ligands with T-type calcium channel blocking activity.

**Results:**

Novel compounds were characterized in radioligand binding assays and *in vitro *functional assays at human and rat CB1 and CB2 receptors. The inhibitory effects of these compounds on transient expressed human T-type calcium channels were examined in tsA-201 cells using standard whole-cell voltage clamp techniques, and their analgesic effects in response to various administration routes (intrathecally, intraplantarly, intraperitoneally) assessed in the formalin model. A series of compounds were synthesized and evaluated for channel and receptor activity. Compound NMP-7 acted as non-selective CB1/CB2 agonist while NMP4 was found to be a CB1 partial agonist and CB2 inverse agonist. Furthermore, NMP-144 behaved as a selective CB2 inverse agonist. All of these three compounds completely inhibited peak Cav3.2 currents with IC_50 _values in the low micromolar range. All compounds mediated analgesic effects in the formalin model, but depending on the route of administration, could differentially affect phase 1 and phase 2 of the formalin response.

**Conclusions:**

Our results reveal that a set of novel cannabinioid receptor ligands potently inhibit T-type calcium channels and show analgesic effects *in vivo*. Our findings suggest possible novel means of mediating pain relief through mixed T-type/cannabinoid receptor ligands.

## Background

Cannabinoid (CB) receptors are the members of G protein-coupled receptor (GPCR) superfamily. They can be activated by the phytocannabinoid Δ9-tetrahydrocannabinol (Δ9-THC) and endogenous cannabinoids, such as anandamide and 2-arachidonyl glycerol (2-AG) (for review, see [1]). To date, two members of the CB receptor family have been identified, namely CB1 and CB2 receptors [2,3]. CB1 receptors are mainly expressed in the central nervous system and peripheral neurons. They are coupled to the G_i/o _pathway and act on effectors such as A-type and inwardly rectifying potassium channels [4-6], as well as N- and P/Q-type calcium channels [5,7,8]. Application of CB1 agonists can inhibit the release of a number of neurotransmitters, which in turn, can mediate cognitive and psychotropic effects [9], impair motor function and induce analgesic effects [10]. CB2 receptors were originally identified in the peripheral immune system, where their activation modulates the cell migration and cytokine release via G_i/o _signaling (for review, see [11,12]). Recently, several studies have shown that the expression of CB2 receptors in microglia is increased during inflammation [13,14], and that CB2 receptors are upregulated in peripheral nerve fibers and spinal cord sensory neurons following nerve injury [15-17]. In addition, a number of CB2-selective ligands have been shown to possess anti-nociceptive effects in various animal pain models, indicating an important role of CB2 receptors in nociceptive signaling [18-20].

T-type voltage-gated calcium channels are another key mediator in pain signaling [21-25]. T-type channels are highly expressed in certain subsets of primary afferent pain fibers, where they can initiate the action potential firing and the generation of burst firing. Intrathecal inhibition of T-type channels with ethosuximide [26] or knockdown of a specific T-type channel subtype, Cav3.2, by antisense depletion induces potent analgesic effects in rodents [27]. Interestingly, several endocannabinoids (anandamide and its methyl derivatives and N-arachidonoyl dopamine) [28-30] and phytocannabinoids (Δ9-tetrahydrocannabinol and cannabidiol) [31] can directly block T-type calcium channels with potencies in the high nanomolar and low micromolar range, and can trigger analgesia when delivered directly into the hindpaw [30]. Notably, these peripheral effects were abolished in a Cav3.2 channel KO mouse.

In this study, we synthesized and pharmacologically characterized a series of novel cannabinoid CB1/CB2 receptor ligands (NMP compounds). We screened the series of CB ligands for T-type channel blocking activity, and then tested their analgesic effects in an *in vivo *model of inflammatory pain. Our data show that mixed T-type/CB ligands may provide a new strategy for developing effective pain therapeutics.

## Results

### In vitro characterization of NMP compounds

**Figure 1:**
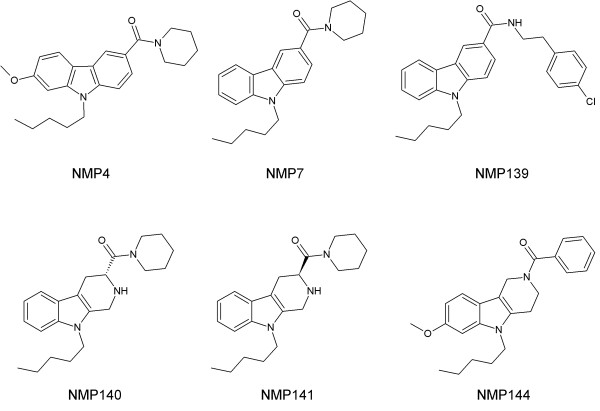
**NMP compounds selected for this study**. Carbazole derivatives NMP-4, NMP-7 and NMP-139, β-carboline derivatives NMP-140 and NMP-141 and γ-carboline derivative NMP-144 were selected and used in this study.

In order to identify compounds potentially interacting with cannabinoid receptors, a set of tricyclic compounds (Figure 1) was selected from our compound library based on carbazole and carboline scaffolds. These compounds were tested for their cannabinoid activities but also on serotonin receptors 5-HT2A and 5-HT2C to discard any promiscuous ligands. In the primary binding assays all of the compounds except NMP-139 displaced more than 50% of [^3^H]CP55,490 in HEK293 cells expressing human CB2 receptor, and in rat brain homogenates expressing CB1 receptors (Table 1). These results were confirmed in competition binding assays (Table 2). In contrast, none of the compounds significantly displaced [^3^H]ketanserin and [^3^H]mesulergine in HEK293 cells expressing human 5-HT2A or rat 5-HT2C receptors, suggesting lack of 5HT receptor activity and discarding GPCR promiscuous activities. Binding studies for NMP-7 were not performed since the functional assay data were already available for this compound (Table 2). NMP-4 exhibited the best affinities for both CB1 and CB2 receptors with *Ki *values of respectively 12.8 nM at rat CB1 receptors and 7.5 nM at human CB2 receptors (Table 2). Functional activities confirm these results since EC_50 _values of NMP-4 in GTPγ[^35^S] functional assays were 118.3 nM at human CB1 with an efficacy of 30.4%, and 9.8 nM with an efficacy of -76.4% at human CB2. These data indicate that NMP-4 acts as a CB1 agonist and a CB2 inverse agonist.

**Table 1:** Primary radioligand competitive binding assays

Ligand (10 μM)	Human 5-HT2A (%)	Rat 5-HT2C (%)	Rat CB1 (%)	Human CB2 (%)
NMP4	23.9	-9.3	99.3	104.4

NMP7	ND	ND	ND	ND

NMP139	7.7	-7.8	46.3	51.3

NMP140	11.2	-4.2	79.3	71.1

NMP141	11.9	-8.4	88.6	88.7

NMP144	-5.2	13	67.4	90.6

ND: not determined

**Table 2:** Radioligand competitive binding assays and GTPγ[^35^S] functional activity.

	Mean K_i _(nM)		GTPγ[^35^S] functional assays			
	
Ligand	Rat CB1	Human CB2	Human CB1		Human CB2	
			
			EC_50 _(nM)	E_max _(%)	EC_50 _(nM)	E_max _(%)
NMP4	12.8 ± 1.8	7.5 ± 0.7	118.3 ± 4	30.4	9.8 ± 0.3	-76.4

NMP7	ND	ND	96.9 ± 11.9	73.6	10.5 ± 1.8	30.8

NMP139	NA	3594 ± 424	ND	ND	ND	ND

NMP140	1252 ± 290	2874 ± 515	ND	ND	ND	ND

NMP141	906.2 ± 211.3	480.7 ± 52.9	NA	NA	112.4 ± 3.3	51.9

NMP144	1143 ± 264	706.2 ± 110.1	NA	NA	35.7 ± 13.9	-48.1

ND: not determined; NA: not active at 1 μM

EC_50 _values for the carbazole analog NMP-7 were respectively 96.9 nM at human CB1 with an efficacy of 73.6%, and 10.5 nM with an efficacy of 30.8% at human CB2 indicating that NMP-7 behaves as a CB1 and CB2 agonist. Functional activities for the β-carboline derivative NMP-140 were not performed because of the low affinities at CB1 and CB2 determined in the binding studies. The corresponding eutomer NMP-141 did not show any agonistic or antagonistic activities at human CB1 receptors in the GTPγ[^35^S] assay. EC_50 _value of NMP-141 for CB2 receptor was 112.4 nM, with an efficacy of 51.9%. Hence, NMP-141 appears to be a selective CB2 agonist. In contrast, the γ-carboline derivative NMP-144 appears to behave as a CB2 inverse agonist with a CB2 EC_50 _value of 35.7 and an efficacy of -48.1%.

Overall, our results indicate that tricyclic scaffolds such as carbazole or carboline can exhibit high affinities for the cannabinoid receptors CB1 and CB2. As we previously demonstrated [32], we were able to reverse agonist activity to inverse agonist activity by adding a methoxy moiety in these scaffolds (NMP-4 and NMP-7).

### NMP compounds block T-type calcium channels

**Figure 2:**
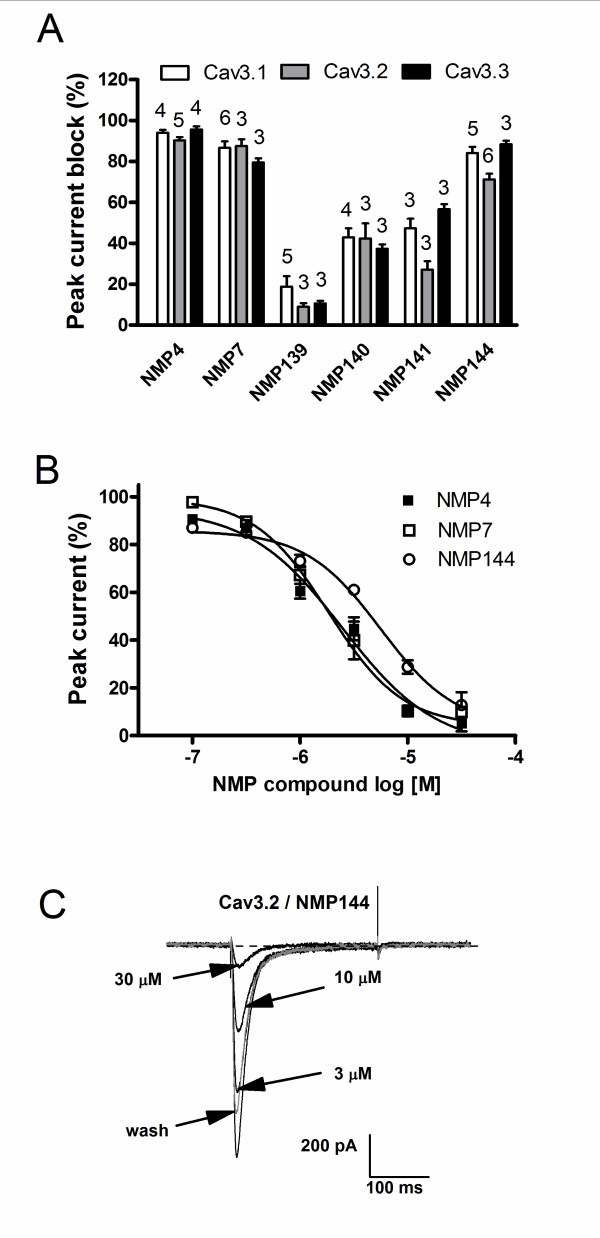
**Inhibition of T-type calcium channels by NMP compounds.  **. (A) The histogram summarizes the inhibitory effects of 10 μM NMP compounds on three subtypes of recombinant T-type calcium channels expressed in tsA-201 cells. The number of cells that have been tested is indicated on the top of the bars. (B) Further characterization of compounds NMP-4, NMP-7 and NMP-144 on Cav3.2 channels. The IC_50 _values obtained from the fit to the dose response relation were 2.47 μM, 1.84 μM 5.59 μM, respectively, for Cav3.1, Cav3.2 and Cav3.3. Data represent the mean ± SEM from 3 - 8 cells of each concentration of compounds. (C) Representative traces from a single cell showing the inhibitory effects of NMP-144 on Cav3.2 with different concentrations and recovery upon washout.

Studies have shown that several endocannabinoids and phytocannabinoids have direct inhibitory actions on T-type voltage-gated calcium channels [28-31]. To determine whether the NMP compounds produced a similar effect, we characterized their actions on transiently expressed T-type calcium channels via whole-cell patch-clamp recordings. Cells were held at -100 mV and barium current was evoked by a depolarizing pulse to -20 mV. As seen in Figure 2A, the initial screen with 10 μM of each compounds revealed a varying degree of inhibition of T-type calcium channels, however, for each compound, the degree of inhibition was similar for all three Cav3 isoforms. The two carbazole derivatives NMP-4 and NMP-7 exhibited potent inhibition of T-type current (80 ~ 95%), which could be rapidly reversed by wash-out. In contrast, NMP-139, an analogue of NMP-4 and NMP-7 that was inactive at CB1/CB2 receptors, yielded minimal inhibition on T-type current (< 20%). Among those carboline derivatives, NMP-144 (γ-carboline derivative, a selective CB2 inverse agonist) displayed robust block of T-type current (~ 70 to 90%), but had only a small effect on N-type calcium channels (20%, n = 5).

To further characterize compounds NMP-4, NMP-7 and NMP-144, we investigated the concentration dependence of their action on Cav3.2, the major T-type calcium channel subtype implicated in the afferent pain pathway (Figure 2B, C). Concentration-response curves revealed that NMP-4 and NMP-7 have similar IC_50_s (2.47 μM and 1.84 μM, respectively) and are approximately 2-fold more potent in blocking T-type current compared to NMP-144 (IC_50 _= 5.59 μM).

**Figure 3:**
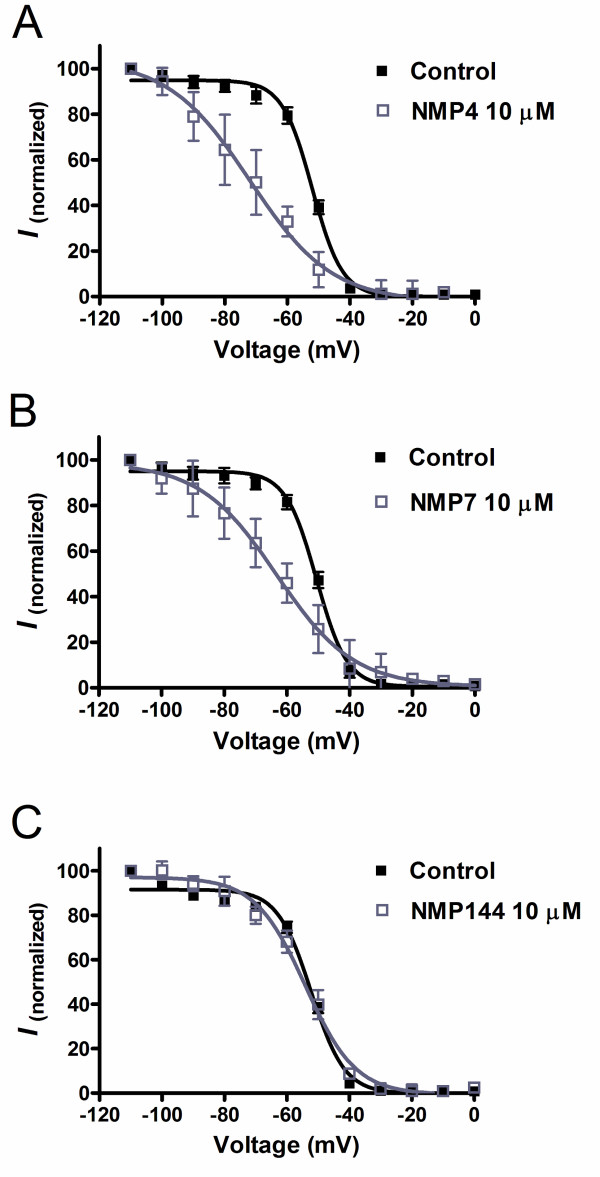
**Steady-state inactivation curves obtained from Cav3.2 channels before and after application of 10 μM NMP-4 (A), NMP-7 (B) or NMP-144 (C)**. The half-inactivation potentials before and after the treatment with drugs were as follows: NMP-4 -52.9 ± 1.4 mV and -72.9 ± 4.7 mV (n = 4, *P *< 0.01, paired *t *test); NMP-7: -50.5 ± 0.6 mV and -63.3 ± 3.5 mV (n = 4, *P *< 0.05, paired *t *test); NMP-144: -53.7 ± 1.6 mV and -55.8 ± 2.2 mV (n = 4, *P *> 0.05, paired *t *test).

The experiments presented in Figure 2 were conducted at a holding potential of -110 mV and hence reflect tonic T-type channel block. To determine whether these compounds also affected inactivated channels, we recorded steady-state inactivation curves prior and after application of NMP-4, NMP-7 or NMP-144. As shown in Figure 3, application of 10 μM NMP-4 or NMP-7 respectively induced 20 mV and 13 mV hyperpolarizing shifts in the half inactivation potential of Cav3.2 channels (Figure 3A, B), thus resulting in additional inhibition when cells are held at a typical neuronal resting membrane potential. In contrast, the same concentration of NMP-144 did not significantly affect voltage-dependent inactivation (Figure 3C), suggesting that NMP-144 blocks Cav3.2 channels via a distinct mechanism from that of NMP-4 and NMP-7 which differ in their backbones from NMP-144 (see Figure 1). Altogether, our results indicate that a subset of CB1/CB2 ligands can act as potent T-type calcium channel blockers. For characterization of *in vivo *effects, we thus focused on three compounds that shared T-type channel blocking activity, but displayed distinct actions on CB1 and CB2 receptors.

### Effect of NMP-4 or NMP-144 on formalin-induced nociception

**Figure 4:**
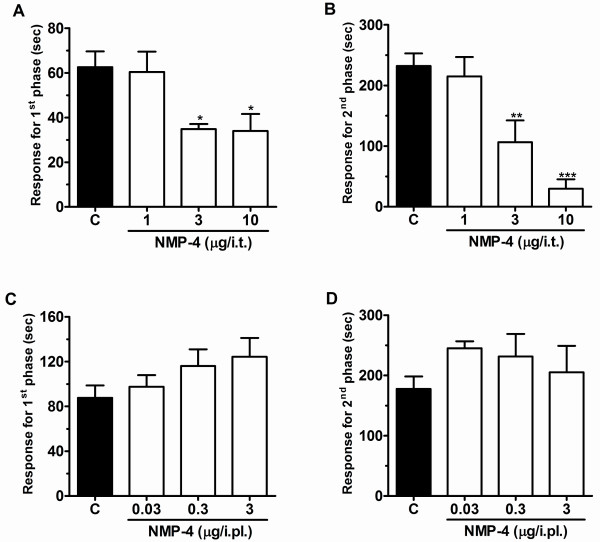
**Effect of NMP-4 administered by i.t. (A and B) or i.pl. (C and D) (co-administered with formalin) routes on the first (A, C) and second (B, D) phases of formalin-induced pain**. Each bar represents the mean responses from 4-7 animals and the error bars indicate the S.E.M. Control values (indicated by "C") are from animals injected with 5% of DMSO and the asterisks denote the significance relative to the control group. *P < 0.05, **P < 0.01, ***P < 0.001. (one-way ANOVA followed by Dunnett' test).

**Figure 5:**
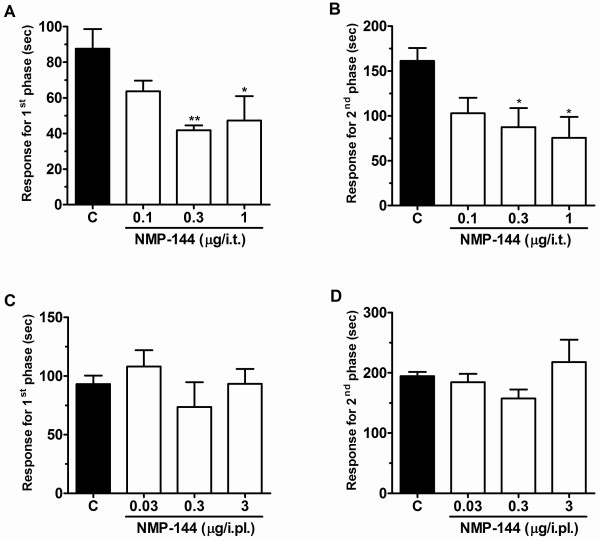
**Effect of NMP-144 administered by i.t. (A and B) or i.pl. (C and D) (co-administered with formalin) routes on first (A, C) and second (B, D) phases of formalin-induced pain**. Each bar represents the mean response of 4-6 animals and the error bars indicate the S.E.M. Control values (indicated by "C") are from animals injected with 5% of DMSO and the asterisks denote the significance relative to the control group. *P < 0.05, **P < 0.01. (one-way ANOVA followed by Dunnett' test).

When delivered spinally 10 minutes prior to behavioural assessment, both NMP-4 **(**1-10 μg/i.t.) and NMP-144 (0.1-1 μg/i.t.) produced significant dose dependent inhibition of both neurogenic (first) and inflammatory (second) phases of formalin-induced nociception (Figures 4 and 5). In the presence of NMP-4 and NMP-144, pain response times (i.e., time spent licking and biting) were reduced, respectively by 44 ± 4% (Figure 4A) and 52 ± 3% (Figure 5A) (first phase) and 87 ± 7% (Figure 4B) and 53 ± 14% (Figure 5B) (for second phase). In contrast, when NMP-4 (0.03-3 μg/paw) or NMP-144 (0.03-3 μg/paw) were administered peripherally through intraplantar coinjection with formalin, no effect was observed (Figures 4C, D and 5C, D). Altogether, these data show that compounds with two completely different CB receptor activity profiles, but overlapping T-type channel blocking activity, block inflammatory pain and mediate analgesia when administered spinally.

### Effect of NMP-7 on formalin-induced nociception

**Figure 6:**
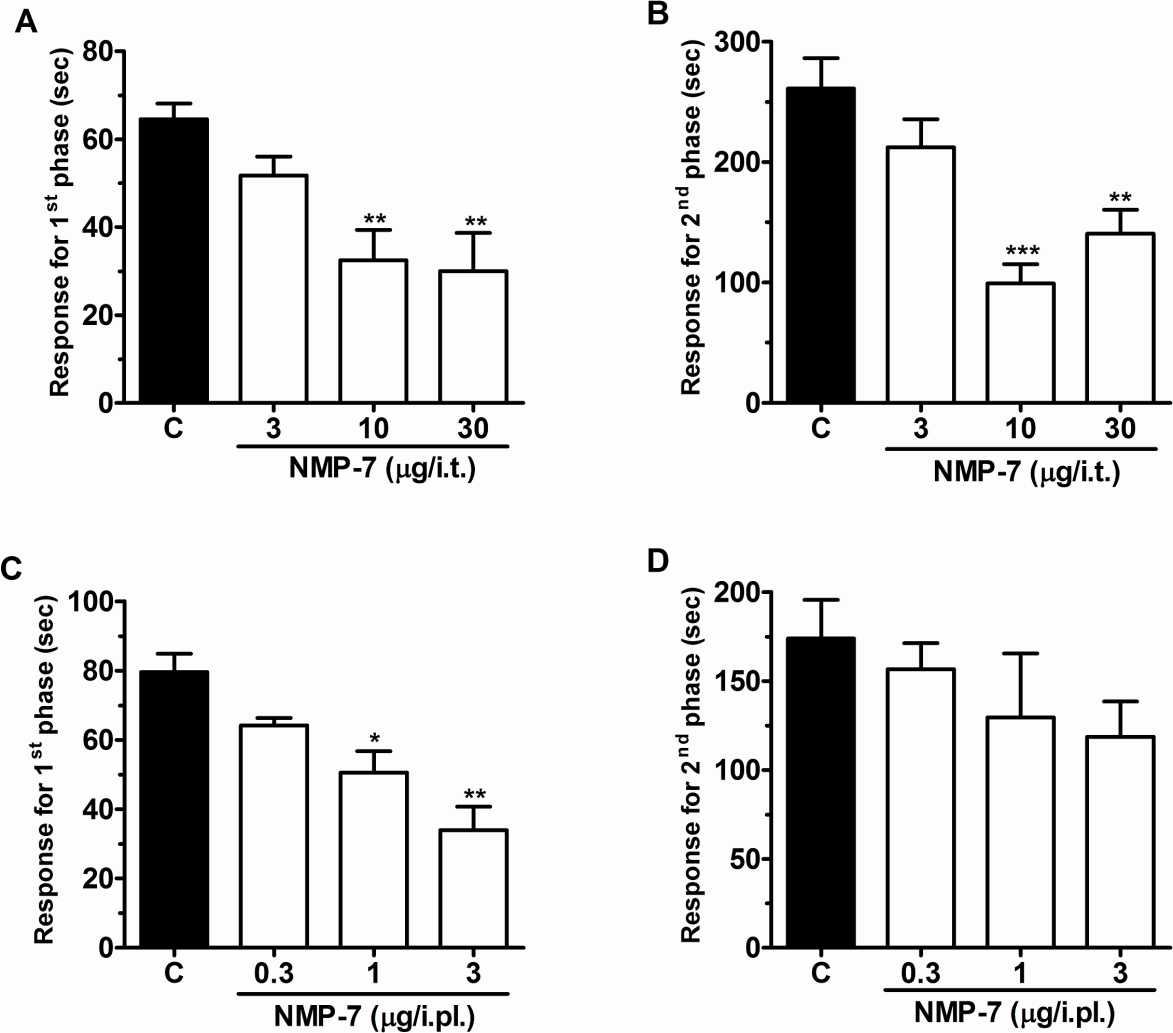
**Effect of NMP-7 administered by i.t. (A and B), or i.pl. (C and D) (co-administered with formalin) routes on first (A, C) and second (B, D) phases of formalin-induced pain**. Each bar represents the mean response from 4-7 animals and the error bars indicate the S.E.M. Control values (indicated by "C") are from animals injected with 5% of DMSO and the asterisks denote the significance relative to the control group. *P < 0.05, **P < 0.01, ***P < 0.001. (one-way ANOVA followed by Dunnett' test).

**Figure 7:**
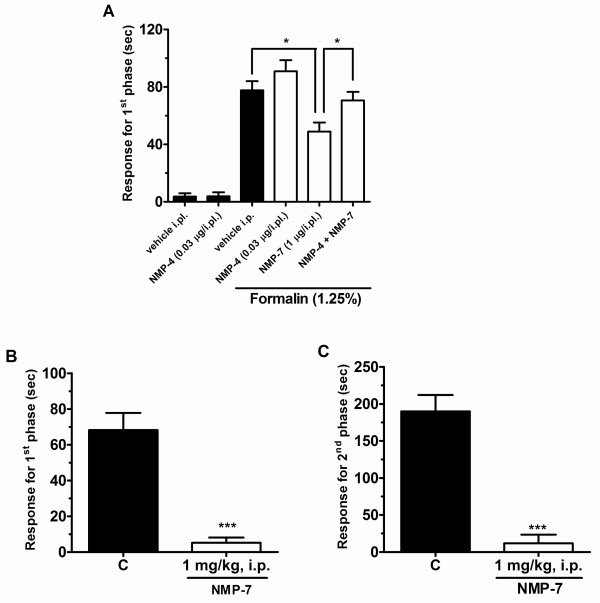
**Effect of i.pl. pre-treatment with NMP-4 (0.03 μg/i.pl.) on the antinociceptive effect of NMP-7 (1 μg/i.pl.) on first phase of formalin-induced pain in mice (A)**. Each bar represents the mean response from 5-7 animals and the error bars indicate the S.E.M. Vehicle values are from animals injected with PBS solution with 5% of DMSO. The asterisks denote the significance level: *P <0.05 (two-way ANOVA followed by the Newman-Keuls' test). B, C. Effect of systemically delivered NMP-7 (1 mg/kg, i.p.) on both phases of formalin induced pain. Each bar represents the mean response from 5-6 animals and the error bars indicate the S.E.M. Control values (indicated by "C") are from animals injected with 5% of DMSO and the asterisks denote the significance relative to the control group. *P < 0.05, **P < 0.01, ***P < 0.001. (one-way ANOVA followed by Dunnett' test).

As with the two other compounds, NMP-7 administered spinally (i.t., 3-30 μg/i.t, 10 min prior) produced significant inhibition of both neurogenic (0-5 min) and inflammatory (15-30 min) phases of formalin-induced nociception (Figure 6A,B). When NMP-7 was administered peripherally (i.pl., 0.3-3 μg/i.pl., co-injected with formalin) it was also able to inhibit the first phase of formalin-induced nociception (Figure 6C,D). As shown in Figure 7A, i.pl. pre-treatment of the CB2 inverse agonist NMP-4 (0.03 μg/i.pl.) significantly antagonized the antinociceptive effect of NMP-7 (1 μg/i.pl.) on the first phase of formalin-induced pain, suggesting that CB2 receptor activity is linked to the analgesic effect of NMP-7 observed for the neurogenic phase.

We then delivered NMP-7 via the intrperitoneal route. As shown in Figures 7B and 7C, systemic delivery (1 mg/kg, 30 min before behavioural assessment) led to a strong inhibition of the pain responses in phase 1 (92 ± 7%) and phase 2 (94 ± 6%). Intraperitoneal administration of NMP-7 (1 mg/kg, i.p.) 30 minutes before the experiment did not alter locomotor activity in the open-field test when compared with control group (data not shown).

Altogether, these data indicate that CB2 agonist activity (perhaps in addition to the ability to block T-type channels) leads to an inhibition of the neurogenic pain response when administered either systemically, or locally at the site of injury.

## Discussion

Ion channels and G protein coupled receptors in the primary afferent pain pathway are key targets for mediating analgesia. Both CB1 and CB2 receptors play important roles in the pain pathway. For example, CB2 receptors are upregulated during neuropathic pain [15]. CB2 receptor activation inhibits mechanical hyperalgesia in inflammatory and neuropathic pain models [33], and mediates analgesia in models of bone cancer when admistered at the spinal level [34]. Similarly, peripheral activation of CB1 receptors is effective in treating neuropathic and inflammatory pain [35]. One of the key endocannabinoids, anandamide, has been shown to not only activate CB receptors, but to also potently inhibit Cav3.2 T-type channels. T-type channel activity is upregulated in various pain states including neuropathic pain, and pharmacological inhibition or antisense depletion of these channels in dorsal root ganglion neurons mediates analgesia (for review see [25]). Hence one may perhaps expect synergistic analgesic properties in CB receptor agonists with T-type channel antagonist activity. Here we have developed several new synthetic CB receptor ligands with various agonist/inverse agonist activity profiles on CB1 and CB2 receptors, and then screened them for T-type channel blocking activity. The observation that several of these ligands mediated robust T-type channel block may suggest that T-type calcium channels and CB receptors may share a common binding pocket structure for cannabinoids and related compounds, similar to what we described recently for CCR2 receptor ligands and Cav3.2 channels [36].

It is interesting to note that NMP-4, NMP-7 and NMP-144 all mediated analgesia, even though these compounds displayed diametrically different actions on CB receptors. While NMP-7 is an agonist for both CB1 and CB2 receptors, NMP-4 is only a partial agonist for CB1 and an inverse agonist of CB2, and NMP-144 is a selective CB2 receptor inverse agonist. One feature shared among all three compounds is their ability to block T-type channels, thus suggesting T-type inhibition as a main contributor to their analgesic properties. Despite being a CB2 inverse agonist and having little CB1 activity, NMP-4 and NMP-144 blocked both the neurogenic and the inflammatory components of the formalin response, however NMP-144 was the most potent and produced analgesia at doses as low as 0.3 μg/i.t. This suggests that its analgesic actions do not involve CB receptors, but likely occur predominantly via its T-type channel blocking activity. In contrast, these compounds did not produce effects when delivered intraplantarly, suggesting that local administration of a T-type channel inhibitor does not result in pain relief.

On the other hand, the mixed CB1/CB2 agonist NMP-7 potently affected both phases after systemic delivery. The effect of NMP-7 in the first phase was abrogated by a CB2 inverse agonist, suggesting that CB2 receptors are likely responsible for the observed local effects. This fits with reports showing that CB2 receptors are expressed at peripheral nerve endings and play a role in the inflammatory pain or neuropathic pain states [15,37], and the observation that local administration of CB2 agonists contribute to analgesia during in inflammatory pain [37-39]. The potent systemic effects of NMP-7 are more difficult to interpret at the mechanistic level, because in addition to affecting spinal and peripheral receptors/channels, this compound may also show CNS penetration. Nonetheless, our data suggest that a mixed CB2 receptor agonist/T-type channel antagonist may be a suitable strategy towards mediating relief from inflammatory pain.

## Conclusion

Altogether, our experiments identify a novel class of mixed CB/T-type channel ligands with potent analgesic properties. The novel pharmacophores may also serve a starting point for the development of new, more potent T-type channel antagonists.

## Materials and methods

### Chemical synthesis

NMP compounds were synthesized at the Core Laboratory for Neuromolecular Production (Figure 1). Full analytical data for NMP compounds are available in Additional File 1.

Carbazole derivatives (NMP-4, NMP-7 and NMP-139) were prepared in a four-step sequence starting with corresponding carbazole which were alkylated and formylated [40,41]. Oxidation of the resulting aldhehyde [42] yielded the corresponding carboxylic acid. The amide analogues were prepared by amidification with the corresponding amines. β-carboline derivatives (NMP-140, NMP-141) were prepared by amidification of commercially available Boc protected carbolines affording the corresponding amide which is alkylated. The Boc group was selectively removed [43] and converted to the corresponding hydrochlorides. γ-Carboline derivative NMP-144, was prepared by methodologies previously employed [44,45]. Pyrido[4,3-β]indole obtained by Fischer indol synthesis was alkylated and the ethoxycarbonyl group was removed. Direct reaction of the amine previously obtained with the corresponding acyl chlorides afforded the desired compounds.

### cDNA constructs

Human Cav3.2 and Cav3.3 were kindly provided by Drs. Arnaud Monteil (CNRS Montpellier) and Terrance Snutch (University of British Columbia), respectively. Cloning of Cav3.1 was described by us previously [46]. The DNA encoding the human CB1 receptor was isolated from a human brain stem cDNA library [47]. Sequencing confirmed that it was identical to GenBank Accession X54937. The coding sequence of the human CB1 receptor was subcloned as a HindIII-XbaI 1.5 kb DNA fragment in the expression vector pCDNA3 and in a bicistronic expression vector. The human CB2 receptor was cloned by PCR using oligonucleotides based on the sequence published by Munro *et al*. [3] with human genomic DNA as template. Sequencing of the resulting clones identified a fragment of 1.1 kb encoding the human cannabinoid 2 receptor, identical to GenBank Accession X74328. The coding sequence of the human CB2 receptor was inserted into bicistronic expression plasmids as a BamHI-NheI fragment and was subcloned as a BamHI-NheI DNA fragment in a BamHI-XbaI expression vector pCDNA3 (Invitrogen). The sequences of human CB2, human 5-HT2A and rat 5-HT2C used in the binding studies are the NCBI Reference Sequence.

### Cell culture and transfection

HEK293 cells and CHO cells were used in the radioligand binding assay while tsA-201 cells were used in the electrophysiological study. Human CB2, human 5-HT2A, rat 5-HT2C used in the binding studies were cloned into pcDNA5.0FRT and cell lines were made using the FlpIn system from Invitrogen. tsA-201 cell culture and transient calcium channel transfection were performed as described previously [36]. In brief, tsA-201 cells were transfected using the calcium phosphate method. Cav3.1, 3.2 and 3.3 α1 subunits were transfected individually with yellow fluorescent protein as a transfection marker.

### In vitro receptor radioligand CB1 and CB2 binding studies

CB1 and CB2 radioligand binding data were obtained using National Institute of Mental Health (NIMH) Psychoactive Drug Screening Program (PDSP) resources as described earlier [48-50]. Compounds were screened in a competitive binding experiment using, respectively, membrane fractions prepared from rat brain homogenate expressing CB1 receptor and HEK293 cells expressing the human CB2 receptor. Experiments were conducted at a range of different concentrations and in duplicate. The competition binding experiment for CB1 and CB2 was performed in 96 well plates containing Standard Binding Buffer (50 mM Tris HCl, 1 mM EDTA, 3 mM MgCl_2_, 5 mg/ml fatty acid-free BSA, pH 7.4). The radioligand was [^3^H]CP55940, and the reference compound was CP55940. A solution of the compound to be tested was prepared as a 1 mg/ml stock in DMSO and then diluted in Standard Binding Buffer by serial dilution. Radioligand was diluted to five times the assay concentration in Standard Binding Buffer. Aliquots (50 μl) of radioligand were dispensed into the wells of a 96-well plate containing 100 μl of Standard Binding Buffer. Then, duplicate 50-μl aliquots of the test and reference compound dilutions were added. Finally, crude membrane fractions of cells were resuspended in 3 ml of chilled Standard Binding Buffer and homogenized by several passages through a 26 gauge needle, then 50 μl were dispensed into each well. The 250-μl reactions were incubated at room temperature for 1.5 hours, and then harvested by rapid filtration onto Whatman GF/B glass fiber filters pre-soaked with 0.3% polyethyleneimine using a 96-well Brandel harverster. Four rapid 500-μl washes were performed. Filters were placed in 6-ml scintillation tubes and allowed to dry overnight. Bound radioactivity was harvested onto 0.3% polyethyleneimine-treated, 96-well filter mats using a 96-well Filtermate harvester. The filter mats were dried, then scintillant was melted onto the filters and the radioactivity retained on the filters counted in a Microbeta scintillation counter. Raw data (dpm) representing total radioligand binding (i.e., specific + non-specific binding) were plotted as a function of the logarithm of the molar concentration of the competitor (i.e., test or reference compound). Non-linear regression of the normalized (i.e., percent radioligand binding compared to that observed in the absence of test or reference compound) raw data was performed in Prism 4.0 (GraphPad Software) using the built-in three parameter logistic model describing ligand competition binding to radioligand-labeled sites: y = bottom + [(top-bottom)/(1 + 10 × -logIC_50_)] where bottom equals the residual radioligand binding measured in the presence of 10 μM reference compound (i.e., non-specific binding) and top equals the total radioligand binding observed in the absence of competitor. The log IC_50 _(i.e., the log of the ligand concentration that reduces radioligand binding by 50%) is thus estimated from the data and used to obtain the K_i _by applying the Cheng-Prusoff approximation: K_i _= IC_50_/(1 + [ligand]/K_D_) where [ligand] equals the assay radioligand concentration and K_D _equals the affinity constant of the radioligand for the target receptor.

### 5-HT2A and 5-HT2C binding

Radioligand binding data were obtained using National Institute of Mental Health's Psychoactive Drug Screening Program (NIMH/PDSP) using the same protocol described for CB1 and CB2 binding experiments [48-50]. Transfected HEK293 (human 5-HT2A or rat 5-HT2C) cells were used. The radioligands were [^3^H]ketanserin (0.5 nM) and [^3^H]mesulergine (0.5 nM) for respectively 5-HT2A and 5-HT2C experiments. Non-specific binding was determined in the presence of 10 μM chlorpromazine for both 5-HT2A and 5-HT2C binding experiments. Cells were harvested and homogenized in 50 mM Tris HCl, 10 mM MgCl_2_, 0.1 mM EDTA, pH 7.4. Incubation of radioreceptor binding assay mixtures was 1.5 hours at room temperature in the dark.

### GTPγ[^35^S] functional assays

Functional activity was evaluated using GTPγ[^35^S] assay in CHO cell membrane extracts expressing recombinant human CB1 or CB2 receptors as we previously described [51]. Compounds were solubilized in 100% DMSO at a concentration of 10 mM within 4 hours of the first testing session. A predilution for the dose response curve was performed in 100% DMSO and then diluted 100 fold in assay buffer at a concentration 2 fold higher than the concentration to be tested. Compounds were tested for agonist activities in duplicate with CP55,940 (Tocris, Bioscience, Ellisville, MI, USA) as reference agonist. Membranes were mixed with GDP diluted in assay buffer to give 30 μM solution (volume:volume) and incubated for at least 15 min on ice. In parallel, GTPγ[^35^S] (GE Healthcare, Catalogue number SJ1308) were mixed with the beads (PVT-WGA (GE Healthcare, RPNQ001)), diluted in assay buffer at 50 mg/ml (0.5 mg/10 μl) (volume:volume) just before starting the reaction. The following reagents were successively added in the wells of an Optiplate (Perkin Elmer): 50 μl of ligand, 20 μl of the membranes:GDP mix, 10 μl of assay buffer for agonist testing, and 20 μl of the GTPγ[^35^S]:beads mix. The plates were covered with a topseal, shacked on an orbital shaker for 2 min, and then incubated for 1 hour at room temperature. Then the plates were centrifuged for 10 min at 2000 rpm and counted for 1 min/well with a PerkinElmer TopCount reader. Assay reproducibility was monitored by the use of reference compound CP 55,940. For replicate determinations, the maximum variability tolerated in the test was of ± 20% around the average of the replicates. Efficacies (*E_max_*) for CB1 or CB2 are expressed as a percentage relative to the efficacy of CP 55,940.

### Electrophysiology

Whole-cell currents were recorded from tsa-201 cells 2-4 days after transfection. The external recording solution contained (in mM): BaCl_2 _(20), MgCl_2 _(1), HEPES (10), TEA-Cl (40), CsCl (65), d-glucose (10) and pH 7.2 adjusted with TEA-OH. The internal pipette solution was composed of (in mM) Cs-methanesulfonate (108), MgCl_2 _(4), EGTA (9), HEPES (9) and pH 7.2 adjusted with CsOH. The internal solution was supplemented with 0.6 mM GTP (sodium salt) and 2 mM ATP (tris salt), which were added directly to the internal solution immediately prior to use. Pipettes with a resistance of 2~5 MΩ when filled with internal solution were used for recording. Currents were elicited from a holding potential of -100 mV and were measured by conventional whole-cell patch clamp using an Axopatch 200B amplifier in combination with Clampex 9.2 software (Axon Instruments, Foster City, CA). Data were filtered at 1 kHz (8-pole Bessel) and digitized at 10 kHz with a Digidata 1320 (Axon Instruments, Foster City, CA). Series resistance were compensated by 85% in all experiments. An online P/4 programme was used to subtract the leak currents. Drugs were dissolved in DMSO at the following stock concentrations: 20 mM for NMP-4 and NMP-7, and 10 mM for NMP-139, NMP-140, NMP-141 and NMP-144. Prior to the experiment, drugs were diluted into external recording solution with a final DMSO concentration no higher than 0.3%. DMSO was also included in the control solution. Drug delivery was controlled by a Valvelink8.2 fast perfusion system (Automate Scientific Inc., Berkeley, CA). The perfusion tip was positioned a few hundred microns from the cell and kept as constant as possible throughout the experiments. Electrophysiological data were analyzed using Clampfit 9.2 (Axon Instruments, Foster City, CA) and GraphPad Prism 5 software (GraphPad Software Inc., San Diego, CA). Concentration-response curves for compound inhibition were generated using Hill equation I/I_control _= 1/[1 + (IC_50_/[compound])^n^], where I is the normalized current at a given concentration of the compound, IC_50 _is the concentration of the compound yielding a current that is half of the control current, I_control_, and n is the Hill coefficient. Steady-state inactivation curves were fitted using the Boltzmann equation: *I *= 1/(1 + *e *^(V-Vh)/*k*^), where V_h _is the half inactivating potential and k is the slope factor.

### Animals

All experiments were conducted following the protocol approved by the Institutional Animal Care and Use Committee (protocol #M09130) and all efforts were made to minimize animal suffering. Male mice of C57BL/6J strain weighing 25~30 g, 10 weeks old were used. Animals were housed at a maximum number of five per cage (30 × 20 × 15 cm) with food and water *ad libitum*. They were kept in 12 h light/dark cycles (lights on at 7:00 a.m.) at a temperature of 23 ± 1°C. All manipulations were carried out between 11:00 am and 3:00 pm. All drugs were dissolved in DMSO in amount that did not exceed a final DMSO concentration of 5%. Control animals received the same vehicle used to dilute the compounds. When drugs were delivered by intraperitoneal (i.p.) route, a constant volume of 10 ml/kg body weight was injected. When drugs were administered by intrathecal (i.t.) or intraplantar (i.pl.) routes, respectively volumes of 5 μl or 20 μl were injected. Appropriate vehicle-treated groups were also assessed simultaneously.

### Formalin Test

The formalin test is a widely used model that allows us to evaluate two different types of pain: neurogenic pain (phase 1) is caused by direct activation of nociceptive nerve terminals, while inflammatory pain (phase 2) is mediated by a combination of peripheral input and spinal cord sensitization [52,53]. Animals received 20 μl of a formalin solution (1.25%) made up in PBS injected intraplantarly (i.pl.) in the ventral surface of the right hindpaw. Following i.pl. injection of formalin, the animals were immediately placed individually into observation chambers and the time spent licking or biting the injected paw was be recorded and considered as nociceptive response. We observed animals individually from 0-5 min (neurogenic phase) and 15-30 min (inflammatory phase).

### Intrathecal injections

Intrathecal injections were given to fully conscious mice using the method previously described by Hylden and Wilcox [54]. Briefly, the animals were manually restrained, and a 30-gauge needle connected by a polyethylene tube to a 25 μl Hamilton syringe (Hamilton, Birmingham, UK) was inserted through the skin and between the vertebrae into the subdural space of the L5-L6 spinal segments. Intrathecal injections were given over a period of 5 seconds.

### Statistical analysis

The results for all the experiments are presented as mean ± S.E.M. The statistical significance of differences between groups was detected by ANOVA followed by Dunnett's or Newman-Keuls' test when appropriate. *P*-values less than 0.05 were considered significant.

## Competing interests

The authors declare that they have no competing interests.

## Authors' contributions

HY, VMG and RRP performed experiments and analyzed data. GWZ and PD designed experiments, HY, VMG, RRP, GWZ and PD wrote the manuscript. The authors read and approved the final manuscript.

## Supplementary Material

Additional file 1 **mass spectrometry analyses of compounds used in this study**. This file contains raw mass spectrometry analysis data for the various compounds examined in this study. There is also a brief paragraph on LC-MS methodology and a summary of retention time and molecular weights of the individual compounds. Click here for file

## References

[B1] Pertwee RG Howlett AC Abood ME Alexander SP Di Marzo V Elphick MR Greasley PJ Hansen HS Kunos G Mackie K International Union of Basic and Clinical Pharmacology. LXXIX. Cannabinoid receptors and their ligands: beyond CB and CB Pharmacol Rev 2010 62 588 631 10.1124/pr.110.003004 21079038 PMC2993256

[B2] Matsuda LA Lolait SJ Brownstein MJ Young AC Bonner TI Structure of a cannabinoid receptor and functional expression of the cloned cDNA Nature 1990 346 561 564 10.1038/346561a0 2165569

[B3] Munro S Thomas KL Abu-Shaar M Molecular characterization of a peripheral receptor for cannabinoids Nature 1993 365 61 65 10.1038/365061a0 7689702

[B4] Henry DJ Chavkin C Activation of inwardly rectifying potassium channels (GIRK1) by co-expressed rat brain cannabinoid receptors in Xenopus oocytes Neurosci Lett 1995 186 91 94 10.1016/0304-3940(95)11289-9 7777206

[B5] Mackie K Lai Y Westenbroek R Mitchell R Cannabinoids activate an inwardly rectifying potassium conductance and inhibit Q-type calcium currents in AtT20 cells transfected with rat brain cannabinoid receptor J Neurosci 1995 15 6552 6561 7472417 10.1523/JNEUROSCI.15-10-06552.1995 PMC6578016

[B6] McAllister SD Griffin G Satin LS Abood ME Cannabinoid receptors can activate and inhibit G protein-coupled inwardly rectifying potassium channels in a xenopus oocyte expression system J Pharmacol Exp Ther 1999 291 618 626 10525080

[B7] Mackie K Hille B Cannabinoids inhibit N-type calcium channels in neuroblastoma-glioma cells Proc Natl Acad Sci USA 1992 89 3825 3829 10.1073/pnas.89.9.3825 1315042 PMC525583

[B8] Felder CC Joyce KE Briley EM Mansouri J Mackie K Blond O Lai Y Ma AL Mitchell RL Comparison of the pharmacology and signal transduction of the human cannabinoid CB1 and CB2 receptors Mol Pharmacol 1995 48 443 450 7565624

[B9] Lichtman AH Martin BR Delta 9-tetrahydrocannabinol impairs spatial memory through a cannabinoid receptor mechanism Psychopharmacology (Berl) 1996 126 125 131 10.1007/BF02246347 8856831

[B10] Martin BR Lichtman AH Cannabinoid transmission and pain perception Neurobiol Dis 1998 5 447 461 10.1006/nbdi.1998.0218 9974177

[B11] Howlett AC Barth F Bonner TI Cabral G Casellas P Devane WA Felder CC Herkenham M Mackie K Martin BR International Union of Pharmacology. XXVII. Classification of cannabinoid receptors Pharmacol Rev 2002 54 161 202 10.1124/pr.54.2.161 12037135

[B12] Pertwee RG Pharmacological actions of cannabinoids Handb Exp Pharmacol 2005 1 51 10.1007/3-540-26573-2_1 16596770

[B13] Benito C Kim WK Chavarria I Hillard CJ Mackie K Tolon RM Williams K Romero J A glial endogenous cannabinoid system is upregulated in the brains of macaques with simian immunodeficiency virus-induced encephalitis J Neurosci 2005 25 2530 2536 10.1523/JNEUROSCI.3923-04.2005 15758162 PMC6725174

[B14] Maresz K Carrier EJ Ponomarev ED Hillard CJ Dittel BN Modulation of the cannabinoid CB2 receptor in microglial cells in response to inflammatory stimuli J Neurochem 2005 95 437 445 10.1111/j.1471-4159.2005.03380.x 16086683

[B15] Zhang J Hoffert C Vu HK Groblewski T Ahmad S O'Donnell D Induction of CB2 receptor expression in the rat spinal cord of neuropathic but not inflammatory chronic pain models Eur J Neurosci 2003 17 2750 2754 10.1046/j.1460-9568.2003.02704.x 12823482

[B16] Wotherspoon G Fox A McIntyre P Colley S Bevan S Winter J Peripheral nerve injury induces cannabinoid receptor 2 protein expression in rat sensory neurons Neuroscience 2005 135 235 245 10.1016/j.neuroscience.2005.06.009 16084654

[B17] Romero-Sandoval A Eisenach JC Spinal cannabinoid receptor type 2 activation reduces hypersensitivity and spinal cord glial activation after paw incision Anesthesiology 2007 106 787 794 10.1097/01.anes.0000264765.33673.6c 17413917

[B18] Ibrahim MM Porreca F Lai J Albrecht PJ Rice FL Khodorova A Davar G Makriyannis A Vanderah TW Mata HP Malan TP Jr CB2 cannabinoid receptor activation produces antinociception by stimulating peripheral release of endogenous opioids Proc Natl Acad Sci USA 2005 102 3093 3098 10.1073/pnas.0409888102 15705714 PMC549497

[B19] Malan TP Ibrahim MM Deng H Liu Q Mata HP Vanderah T Porreca F Makriyannis A CB2 cannabinoid receptor-mediated peripheral antinociception Pain 2001 93 239 245 10.1016/S0304-3959(01)00321-9 11514083

[B20] Yao BB Hsieh GC Frost JM Fan Y Garrison TR Daza AV Grayson GK Zhu CZ Pai M Chandran P In vitro and in vivo characterization of A-796260: a selective cannabinoid CB2 receptor agonist exhibiting analgesic activity in rodent pain models Br J Pharmacol 2008 153 390 401 10.1038/sj.bjp.0707568 17994110 PMC2219533

[B21] Altier C Zamponi GW Targeting Ca2+ channels to treat pain: T-type versus N-type Trends Pharmacol Sci 2004 25 465 470 10.1016/j.tips.2004.07.004 15559248

[B22] Marger F Gelot A Alloui A Matricon J Ferrer JF Barrere C Pizzoccaro A Muller E Nargeot J Snutch TP T-type calcium channels contribute to colonic hypersensitivity in a rat model of irritable bowel syndrome Proc Natl Acad Sci USA 2011 108 11268 11273 10.1073/pnas.1100869108 21690417 PMC3131334

[B23] Orestes P Todorovic SM Are neuronal voltage-gated calcium channels valid cellular targets for general anesthetics? Channels (Austin) 2010 4 518 522 10.4161/chan.4.6.12873 PMC3021099 21164281

[B24] Park J Luo ZD Calcium channel functions in pain processing Channels (Austin) 2010 4 510 517 10.4161/chan.4.6.12869 PMC3052250 21150297

[B25] Zamponi GW Lewis RJ Todorovic SM Arneric SP Snutch TP Role of voltage-gated calcium channels in ascending pain pathways Brain Res Rev 2009 60 84 89 10.1016/j.brainresrev.2008.12.021 19162069 PMC2692704

[B26] Matthews EA Dickenson AH Effects of ethosuximide, a T-type Ca(2+) channel blocker, on dorsal horn neuronal responses in rats Eur J Pharmacol 2001 415 141 149 10.1016/S0014-2999(01)00812-3 11274992

[B27] Bourinet E Alloui A Monteil A Barrere C Couette B Poirot O Pages A McRory J Snutch TP Eschalier A Nargeot J Silencing of the Cav3.2 T-type calcium channel gene in sensory neurons demonstrates its major role in nociception Embo J 2005 24 315 324 10.1038/sj.emboj.7600515 15616581 PMC545807

[B28] Ross HR Gilmore AJ Connor M Inhibition of human recombinant T-type calcium channels by the endocannabinoid N-arachidonoyl dopamine Br J Pharmacol 2009 156 740 750 10.1111/j.1476-5381.2008.00072.x 19226289 PMC2697747

[B29] Chemin J Monteil A Perez-Reyes E Nargeot J Lory P Direct inhibition of T-type calcium channels by the endogenous cannabinoid anandamide Embo J 2001 20 7033 7040 10.1093/emboj/20.24.7033 11742980 PMC125779

[B30] Barbara G Alloui A Nargeot J Lory P Eschalier A Bourinet E Chemin J T-type calcium channel inhibition underlies the analgesic effects of the endogenous lipoamino acids J Neurosci 2009 29 13106 13114 10.1523/JNEUROSCI.2919-09.2009 19846698 PMC6665211

[B31] Ross HR Napier I Connor M Inhibition of recombinant human T-type calcium channels by Delta9-tetrahydrocannabinol and cannabidiol J Biol Chem 2008 283 16124 16134 10.1074/jbc.M707104200 18390906 PMC3259625

[B32] Diaz P Phatak SS Xu J Astruc-Diaz F Cavasotto CN Naguib M 6-Methoxy-N-alkyl isatin acylhydrazone derivatives as a novel series of potent selective cannabinoid receptor 2 inverse agonists: design, synthesis, and binding mode prediction J Med Chem 2009 52 433 444 10.1021/jm801353p 19115816

[B33] Elmes SJ Jhaveri MD Smart D Kendall DA Chapman V Cannabinoid CB2 receptor activation inhibits mechanically evoked responses of wide dynamic range dorsal horn neurons in naive rats and in rat models of inflammatory and neuropathic pain Eur J Neurosci 2004 20 2311 2320 10.1111/j.1460-9568.2004.03690.x 15525273

[B34] Curto-Reyes V Llames S Hidalgo A Menendez L Baamonde A Spinal and peripheral analgesic effects of the CB2 cannabinoid receptor agonist AM1241 in two models of bone cancer-induced pain Br J Pharmacol 2010 160 561 573 10.1111/j.1476-5381.2009.00629.x 20233215 PMC2931557

[B35] Yu XH Cao CQ Martino G Puma C Morinville A St-Onge S Lessard E Perkins MN Laird JM A peripherally restricted cannabinoid receptor agonist produces robust anti-nociceptive effects in rodent models of inflammatory and neuropathic pain Pain 151 337 344 10.1016/j.pain.2010.07.019 20696525

[B36] You H Altier C Zamponi GW CCR2 receptor ligands inhibit Cav3.2 T-type calcium channels Mol Pharmacol 2010 77 211 217 10.1124/mol.109.059022 19864434

[B37] Hsieh GC Pai M Chandran P Hooker BA Zhu CZ Salyers AK Wensink EJ Zhan C Carroll WA Dart MJ Central and peripheral sites of action for CB receptor mediated analgesic activity in chronic inflammatory and neuropathic pain models in rats Br J Pharmacol 2011 162 428 440 10.1111/j.1476-5381.2010.01046.x 20880025 PMC3031063

[B38] Khasabova IA Chandiramani A Harding-Rose C Simone DA Seybold VS Increasing 2-arachidonoyl glycerol signaling in the periphery attenuates mechanical hyperalgesia in a model of bone cancer pain Pharmacol Res 64 60 67 10.1016/j.phrs.2011.03.007 PMC3104059 21440630

[B39] Nackley AG Zvonok AM Makriyannis A Hohmann AG Activation of cannabinoid CB2 receptors suppresses C-fiber responses and windup in spinal wide dynamic range neurons in the absence and presence of inflammation J Neurophysiol 2004 92 3562 3574 10.1152/jn.00886.2003 15317842

[B40] Bondaletov VGBV Lopatinskii VP Formylcarbazoles. Synthesis and use Izvestiya Vysshikh Uchebnykh Zavedenii, Khimiya i Khimicheskaya Tekhnologiya 1987 30 3 9

[B41] Pajda MBD Fast preparation method of aromatic aldehydes from carbazole and hydroxyaromatic derivatives under microwave irradiation Modern polymeric materials for environmental applications, International Seminar, 2nd,; Krakow, Poland 2006 129 132

[B42] Block MH Boyer S Brailsford W Brittain DR Carroll D Chapman S Clarke DS Donald CS Foote KM Godfrey L Discovery and optimization of a series of carbazole ureas as NPY5 antagonists for the treatment of obesity J Med Chem 2002 45 3509 3523 10.1021/jm011125x 12139462

[B43] Chino NMY Sakakibara S Modification of Trp-residues during the acidolysis of Boc-groups: effect of scavengers Peptide Chemistry 1978 15

[B44] Bonjoch J Diaba F Pages L Perez D Soca L Miralpeix M Vilella D Anton P Puig C Synthesis and structure-activity relationships of gamma-carboline derivatives as potent and selective cysLT(1) antagonists Bioorg Med Chem Lett 2009 19 4299 4302 10.1016/j.bmcl.2009.05.094 19505824

[B45] Khorana N Purohit A Herrick-Davis K Teitler M Glennon RA gamma-Carbolines: binding at 5-HT5A serotonin receptors Bioorg Med Chem 2003 11 717 722 10.1016/S0968-0896(02)00527-8 12538001

[B46] Beedle AM Hamid J Zamponi GW Inhibition of transiently expressed low- and high-voltage-activated calcium channels by trivalent metal cations J Membr Biol 2002 187 225 238 10.1007/s00232-001-0166-2 12163980

[B47] Gerard CM Mollereau C Vassart G Parmentier M Molecular cloning of a human cannabinoid receptor which is also expressed in testis Biochem J 1991 279 Pt 1 129 134 1718258 10.1042/bj2790129 PMC1151556

[B48] Armbruster BN Roth BL Mining the receptorome J Biol Chem 2005 280 5129 5132 15590622 10.1074/jbc.R400030200

[B49] Jensen NH Roth BL Massively parallel screening of the receptorome Comb Chem High Throughput Screen 2008 11 420 426 10.2174/138620708784911483 18673270

[B50] Strachan RT Ferrara G Roth BL Screening the receptorome: an efficient approach for drug discovery and target validation Drug Discov Today 2006 11 708 716 10.1016/j.drudis.2006.06.012 16846798

[B51] Naguib M Diaz P Xu JJ Astruc-Diaz F Craig S Vivas-Mejia P Brown DL MDA7: a novel selective agonist for CB2 receptors that prevents allodynia in rat neuropathic pain models Br J Pharmacol 2008 155 1104 1116 18846037 10.1038/bjp.2008.340 PMC2597252

[B52] Hunskaar S Hole K The formalin test in mice: dissociation between inflammatory and non-inflammatory pain Pain 1987 30 103 114 10.1016/0304-3959(87)90088-1 3614974

[B53] Tjolsen A Berge OG Hunskaar S Rosland JH Hole K The formalin test: an evaluation of the method Pain 1992 51 5 17 10.1016/0304-3959(92)90003-T 1454405

[B54] Hylden JL Wilcox GL Intrathecal morphine in mice: a new technique Eur J Pharmacol 1980 67 313 316 10.1016/0014-2999(80)90515-4 6893963

