# Supplementary material for: Functional characterization and analgesic effects of mixed cannabinoid receptor/T-type channel ligands
Source: Mol Pain. 2011 Nov 17;7:89. doi: 10.1186/1744-8069-7-89 (PMC3250956; doi:10.1186/1744-8069-7-89)
Supplement: Additional file 1 — mass spectrometry analyses of compounds used in this study. This file contains raw mass spectrometry analysis data for the various compounds examined in this study. There is also a brief paragraph on LC-MS methodology and a summary of retention time and molecular weights of the individual compounds. [file 1744-8069-7-89-S1.DOC]

**Functional characterization and analgesic effects of mixed cannabinoid receptor/T-type channel ligands**

**Haitao You^1^, Vinicius M. Gadotti^1^,** **Ravil R. Petrov^2^, Gerald W Zamponi^1^ and Philippe Diaz^2^**

^1^Department of Physiology and Pharmacology, Hotchkiss Brain Institute,

University of Calgary, Calgary, Canada

^2^Core Laboratory for Neuromolecular Production, The University of Montana, Missoula, MT, USA

^1^H NMR of NMP4

in CDCl_3_, Varian 500

^13^C NMR of NMP4

in CDCl_3_, Varian 500

^1^H NMR of NMP7

in CDCl_3_, Varian 500

^13^C NMR of NMP7

in CDCl_3_, Varian 500

^1^H NMR of NMP139

in CDCl_3_, Varian 500

^1^H NMR of NMP141

in CDCl_3_, Varian 500

^13^C NMR of NMP141

in CDCl_3_, Varian 500

^1^H NMR of NMP140

in CDCl_3_, Varian 500

^1^H NMR of NMP144

in CDCl_3_, Varian 500

^1^H NMR of NMP144

in CDCl_3_/MeOH-d_4_, Varian 500

**LC/MS analyses**

LC-MS analyses were performed on a Waters/Micromass LCT, TOF equipped with an Alliance HT Waters 2795 liquid chromatography system and a Waters 2487 dual absorbance detector. The liquid chromatrography conditions were as follow: a Phenomenex Gemini-NX 3-um C18 110A 50x4.60 mm column was used and it was eluted with a gradient made up of two solvent mixtures. Solvent A consisted of water and 0.08% TFA. Solvent C consisted of acetonitrile. The gradient was processed as follows:

Time        A%    B%      C%      D%    Flow (ml/min)

  0.00     90.0     0.0       10.0     0.0     0.400

  6.60     2.0      0.0       98.0     0.0     0.400

  8.00     2.0      0.0       98.0     0.0     0.400

  8.20     90.0     0.0       10.0     0.0     0.400

 9.00     90.0    0.0       10.0     0.0     0.400

 12.00    90.0     0.0       10.0     0.0     0.400

R.T.: Retention time

MS: M+H^+^
